# Is hypoalbuminemia a risk factor for high-dose methotrexate toxicity in children with acute lymphoblastic leukemia?

**DOI:** 10.1186/s43046-022-00122-7

**Published:** 2022-04-18

**Authors:** Shaimaa Barakat, Hala Assem, Mostafa Salama, Neveen Mikhael, Yasmine El Chazli

**Affiliations:** 1 Department of Pediatrics, General Organization for Teaching Hospitals and Institutes, Cairo, Egypt; 2 Department of Pediatrics, Hematology and Oncology Unit, Faculty of Medicine, Alexandria University, Alexandria, Egypt; 3 Department of Clinical and Chemical Pathology, Faculty of Medicine, Alexandria University, Alexandria, Egypt

**Keywords:** Acute lymphoblastic leukemia (ALL), High-dose methotrexate (HDMTX), Toxicities, Pre-infusion albumin

## Abstract

**Background:**

Repeated high-dose methotrexate (HDMTX) is a critical component of contemporary childhood acute lymphoblastic leukemia (ALL) treatment regimens. Serum albumin is considered a carrier of methotrexate (MTX) in the blood. Hypoalbuminemia is not a rare finding in children with leukemia. This study aimed to investigate the relationship between pre-infusion serum albumin and possible HDMTX toxicities.

**Methods:**

Thirty Egyptian children with ALL were consecutively enrolled in the study between May 2018 and July 2020. They were prospectively followed up while receiving HDMTX during the consolidation phase of the TOTAL study XV protocol. HDMTX was administered intravenously as a 24-h infusion every 2 weeks. Doses of 2.5 g/m^2^ were used for low-risk patients and 5 g/m^2^ for standard/high-risk patients. The Common Terminology Criteria for Adverse Events (V.4.03) was used to report the observed toxicities after HDMTX cycles. Plasma MTX levels were estimated at 24 h (MTX_24_) from the beginning of HDMTX infusion in the first consolidation cycle. Serum albumin level was determined before HDMTX administration, and pre-infusion hypoalbuminemia was defined when serum albumin was <3.5 g/dL.

**Results:**

The patients’ age ranged from 2.3 to 13.3 years at diagnosis, and most of them had B cell ALL (86.7%). Overall, 120 HDMTX cycles were analyzed, equally distributed between low and standard/high risk. Grade 3–4 anemia, grades 3–4 thrombocytopenia, febrile neutropenia, and oral mucositis were significantly more frequent in HDMTX cycles with pre-infusion hypoalbuminemia than those with normal pre-infusion albumin (*p*=0.003, *p*=0.007, *p*=0.006, and *p*=0.001, respectively). In addition, pre-infusion hypoalbuminemia was significantly associated with additional hospitalization due to HDMTX toxicity (*p*=0.031). Most HDMTX toxicities were comparable irrespective of the MTX dose. Oral mucositis was more frequently encountered in the 2.5 g/m^2^ than the 5 g/m^2^ HDMTX cycles (46.7 vs. 26.7%, *p*=0.023). A significantly longer hospitalization (due to HDMTX toxicity) was observed in the 5 g/m^2^ HDMTX cycles (median= 7 days vs. 4 days, *p*=0.012).

**Conclusions:**

Serum albumin levels should be checked before starting HDMTX cycles, especially in resource-limited settings where malnutrition is common, and serum MTX monitoring may not be available. Optimizing serum albumin levels before HDMTX may help decrease the possibility of HDMTX toxicities.

## Background

Acute lymphoblastic leukemia (ALL) is the most common cancer in the pediatric age group, and it is responsible for most of cancer-related deaths in children and adolescents [1]. Repeated methotrexate (MTX) courses, administered either via short intravenous (IV) infusion or at high doses (> 1 g/m^2^) over 24 h, followed by folinic acid administration to limit toxic effects, are a critical component of contemporary ALL regimens [2, 3].

Structurally, MTX is a derivative of tetrahydrofolate (the active form of folic acid), which competitively inhibits the main enzymes in folate metabolism, dihydrofolate reductase (DHFR), and thymidylate synthetase (TYMS) [4]. Albumin is alkalotic and binds to weak acids in the serum. As a weak acid, approximately 50% of MTX is bound to serum albumin, making albumin a drug carrier for MTX in the blood [5].

Unfortunately, up to 50% of children with cancer experience hypoalbuminemia before the start of chemotherapy [6]. Hypoalbuminemia can reflect the poor nutritional status as patients with hematologic/lymphatic malignancies and solid metastatic tumors may be cachectic, malnourished, and generally sicker than patients with localized, non-metastatic tumors. In children with ALL, L-asparaginase administered during the induction phase of chemotherapy may cause hypoalbuminemia [7].

It is well known that the nutritional status of children with cancer plays a critical role in the progression of the disease [8]. Malnutrition in children may be defined as a pathological state resulting from inadequate nutrition, including undernutrition and overnutrition (overweight and obesity) [9]. It has been reported that the prevalence of malnutrition in children with ALL from developing countries was higher than those from developed countries (21–52% compared to <10%) [10]. Malnutrition leads to an increased risk of infection and alterations in the drug metabolism with higher toxicity rates, subsequent chemotherapy delays, and possibly treatment cessation [8]. Malnutrition, therefore, can explain the lower survival rates of childhood ALL in developing countries as compared to the developed ones [11].

Management of toxicities related to high-dose methotrexate (HDMTX) continues to be a challenge. Although routine MTX monitoring is recommended during HDMTX therapy, some patients may encounter severe adverse events, even when serum MTX concentrations are within the recommended values. This suggests that plasma MTX concentration is not the only predictive value for adverse clinical events [12]. Moreover, resource-limited settings face practical difficulties in monitoring serum MTX concentrations due to the unavailability of the test and its high cost. So, an economical alternative for predicting HDMTX-related toxicities is needed. The present study aimed to investigate the relationship between pre-infusion albumin level and HDMTX-related toxicities in children with ALL.

## Methods

This study was a prospective cohort in which 30 children with ALL were enrolled (younger than 15 years old at diagnosis). They were admitted to the Pediatric Hematology/Oncology Department, Alexandria University Children’s Hospital, Egypt, for the consolidation phase of chemotherapy between May 2018 and July 2020. The local research ethics committee approved this study. Informed consent/assents were signed by the patient’s legal guardians/patients.

According to the TOTAL XV study protocol, all patients received four HDMTX infusions at 2-week intervals in the consolidation phase [13]. Before HDMTX, pre-hydration (IV) fluids were given for 12 h. Hydration was also continued during HDMTX infusion, and urine pH was maintained above 6.5.

MTX was administered IV as a 24-h infusion (low risk: 2.5 g/m^2^, standard/high risk: 5 g/m^2^); 10% of the dose was administered over 1 h as a loading dose, and the remaining 90% was evenly administered over the following 23 h. Leucovorin (LV) 15 mg/m^2^ IV for standard/high risk or 10 mg/m^2^ IV for low risk was administered 42 h after the start of HDMTX and repeated every 6 h with a total of five doses. Plasma MTX levels were estimated at 24 h (MTX_24_) from the beginning of the HDMTX infusion during the first cycle of consolidation (in 30 HDMTX cycles out of the total 120 cycles) using enzyme-linked immunosorbent assay (ELISA) technique [Human Methotrexate ELISA Kit, SinoGeneClon Biotech Co., Ltd., China]. Triple intrathecal therapy was given on the day of HDMTX in addition to 50 mg/m^2^/day oral 6-mercaptopurine during the 8 weeks of consolidation.

### Toxicity criteria and monitoring

Patients were assessed for possible MTX side effects for a total of 120 HDMTX cycles. Routine laboratory investigations were analyzed before HDMTX and 7 days after drug infusion to detect any possible toxicity. Body mass index (BMI) *Z*-score was calculated using the WHO child growth standards 2006 [14]. Patients were considered underweight if their *Z*-scores were <^_^2 SD and overweight if their *Z*-scores were >2 SD. Those who had 3 SD or more were deemed to be obese. Patients with BMI (from −2 SD to 2 SD) were considered normal. The scoring system for the National Cancer Institute Common Terminology Criteria for Adverse Events (V.4.03) (CTCAE) was used for grading gastrointestinal toxicities (oral mucositis, vomiting, abdominal pain, diarrhea, and upper gastrointestinal hemorrhage), hematological toxicities (neutropenia, anemia, thrombocytopenia, and febrile neutropenia), hepatotoxicity (increased alanine transferase (ALT), aspartate transaminase (AST), and total bilirubin), nephrotoxicity (increased serum creatinine), and toxicities of the nervous system, skin, and eye [15].Additional hospitalizations due to HDMTX toxicity were documented.

Cycles were categorized based on the pre-infusion serum albumin level measured on the day of HDMTX infusion or using the most recent serum albumin recorded within 3 days before infusion. According to the local laboratory reference, serum albumin levels between 3.5 and 5.6 g/dL were considered normal, while hypoalbuminemia was defined when serum albumin levels were <3.5 g/dL [16].

### Statistical analysis

Data were analyzed using IBM SPSS software package version 20.0 (Armonk, NY: IBM Corp). The Kolmogorov-Smirnov test was used to verify the normality of distribution. For comparing categorical variables, a chi-square test was used. Fisher’s exact correction was used when more than 20% of the cells have an expected count of less than 5. Student’s *t* test was used for quantitative variables, to compare between two studied groups. The Mann–Whitney test was used for abnormally quantitative variables, to compare between two studied groups. The significance of the obtained results was judged at the 5% level.

## Results

**Fig. 1:**
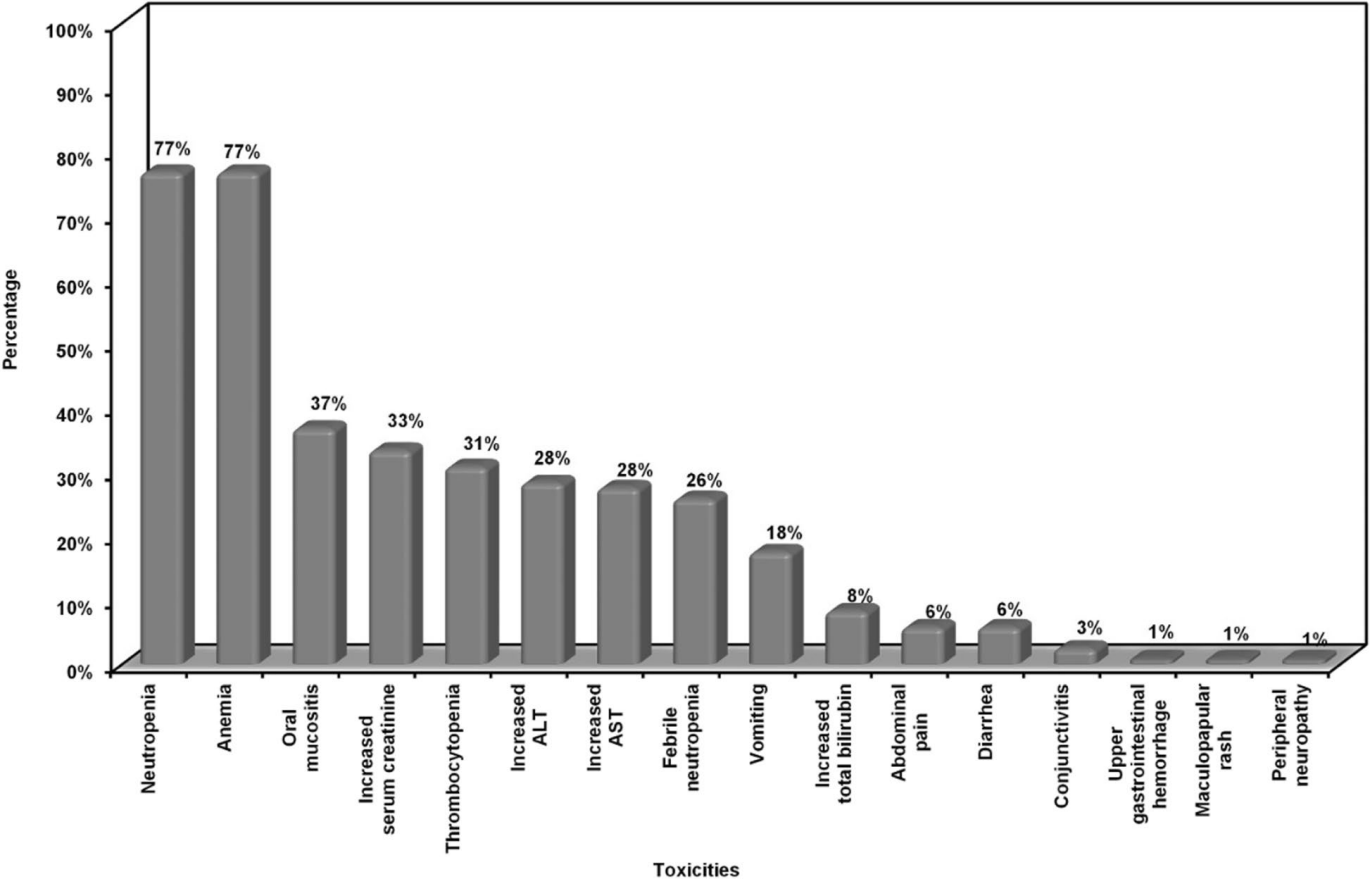
Observed toxicities within the 120 cycles of HDMTX infusions in descending order of frequency. Figure 1 shows the distribution of the observed toxicities within the 120 cycles of HDMTX infusions in descending order of frequency. The most common observed toxicities were hematological toxicities (neutropenia and anemia) that were present in 77% of cycles, each. Oral mucositis came next in frequency 37%, followed by increased creatinine 33%, then thrombocytopenia 31%. Increased ALT, increased AST, and febrile neutropenia were present in 28%, 28%, and 26% of cycles, respectively. Vomiting was reported in 18% of cycles. Increased total bilirubin was present in 8% of cycles. Also, abdominal pain and diarrhea were observed in 6% of cycles. Conjunctivitis was present in 3% of cycles. The least frequently reported toxicities were upper gastrointestinal hemorrhage, maculopapular rash, and peripheral neuropathy (1%) of cycles

The demographic characteristics and clinical features of the included children are presented in Table 1. A total of 120 cycles of HDMTX were analyzed; equally distributed between the low-risk arm (2.5 g/m^2^) and the standard/high-risk arm (5 g/m^2^). The observed toxicities after 120 cycles of HDMTX infusions are shown in Fig. 1 in descending order of frequency. No deaths were attributed to MTX toxicity.

**Table 1:** Demographic and clinical data of included patients

	*N* (%)
Sex	
Male	15 (50.0)
Female	15 (50.0)
ALL subtype	
B-ALL	26 (86.7)
T-ALL	4 (13.3)
Risk stratification	
Low risk	15 (50.0)
Standard/high risk	15 (50.0)
Age at diagnosis (years)	
Min.–Max.	2.30 – 13.30
Median (IQR)	4.25
BMI-for-age *Z*-score	
Underweight (<^_^2 SD)	2 (6.7)
Normal (−2 SD _ 2SD)	22 (73.3)
Overweight (> 2 SD)	4 (13.3)
Obese (> 3 SD)	2 (6.7)

The mean ± SD pre-infusion albumin level of the 120 HDMTX cycles was 3.69 ± 0.58 g/dL (range: 2.5–5 g/dL); 38 (31.7%) HDMTX cycles were associated with pre-infusion hypoalbuminemia.

The relation between pre-infusion albumin (<3.5 g/dL & ≥3.5 g/dL) and the commonly observed clinical and laboratory toxicities are presented in Table 2. We excluded toxicities observed in <20% of cycles from the current analysis. Anemia, thrombocytopenia, febrile neutropenia, and oral mucositis were significantly more frequent in cycles with pre-infusion hypoalbuminemia than cycles with pre-infusion albumin ≥ 3.5 g/dL (*p*=0.024, *p*=0.008, *p*=0.006, and *p*=0.001, respectively). Pre-infusion hypoalbuminemia was significantly associated with grades (G) 3–4 anemia and G3–4 thrombocytopenia (*p*=0.003 and *p*=0.007, respectively).

**Table 2:** Relation between pre-infusion albumin level and commonly observed toxicities of the HDMTX cycles (*N* = 120)

	Pre-infusion Albumin (g/dL)		*p*
	<3.5 g/dL (*N* = 38)	≥3.5 g/dL (*N* = 82)	
	*N* (%)		
Hematological toxicities			
Neutropenia			
No	8 (21.1)	20 (24.4)	“*χ*^2^ test” *p*=0.688
Yes	30 (78.9)	62 (75.6)	
G3–4	24 (63.2)	46 (56.1)	“*χ*^2^ test” *p*=0.466
Anemia			
No	4 (10.5)	24 (29.3)	“*χ*^2^ test” *p*=0.024
Yes	34 (89.5)	58 (70.7)	
G3-4	18 (47.4)	17 (20.7)	“*χ*^2^ test” *p*=0.003
Thrombocytopenia			
No	20 (52.6%)	63 (76.8%)	“*χ*^2^ test” *p*=0.008
Yes	18 (47.4%)	19 (23.2%)	
G3–4	11 (28.9)	8 (9.8)	“*χ*^2^ test” *p*=0.007
Febrile neutropenia^a^			
No	22 (57.9)	67 (81.7)	“*χ*^2^ test” *p*=0.006
Yes	16 (42.1)	15 (18.3)	
Hepatic toxicity			
Increased ALT			
No	26 (68.4)	60 (73.2)	“*χ*^2^ test” *p*=0.591
Yes	12 (31.6)	22 (26.8)	
G3–4	1 (2.6%)	5 (6.1%)	^FE^*p*=0.663
Increased AST			
No	24 (63.2)	63 (76.8)	“*χ*^2^ test” *p*=0.119
Yes	14 (36.8)	19 (23.2)	
G3–4	2 (5.3%)	2 (2.4%)	^FE^*p*=0.590
Gastrointestinal toxicity			
Oral mucositis			
No	16 (42.1)	60 (73.2)	“*χ*^2^ test” *p*=0.001
Yes	22 (57.9)	22 (26.8)	
G3–4	9 (23.7%)	11 (13.4%)	“*χ*^2^ test” *p*=0.160
Nephrotoxicity			
Increased creatinine			
No	22 (57.9)	58 (70.7)	“*χ*^2^ test” *p*=0.165
Yes	16 (42.1)	24 (29.3)	
G3–4	0 (0%)	2 (2.4%)	^FE^*p*=1.000
Hospitalization^b^			
No	23 (60.5)	65 (79.3)	“*χ*^2^ test” *p*=0.031
Yes	15 (39.5)	17 (20.7)	
Length of hospitalization	(*N* = 15)	(*N* = 17)	
Median (IQR)	4 (3–8)	6 (3.5–7.5)	“*U* test” *p*=0.576
Min.–Max.	2–14	2–12	

*P*-value significant when *p* <0.05

^a^There is no grades 1 or 2 in the CTCAE score system grading febrile neutropenia, and it was reported in the present study only in grade 3 [15]

^b^Extra days of hospitalization due to HDMTX-related toxicities

Since pre-infusion hypoalbuminemia was associated with G3–4 anemia, we compared pre-infusion hemoglobin levels between cycles with and without pre-infusion hypoalbuminemia. There was no significant difference between the two groups with a mean (SD) of 10.18±1.31 g/dL and 9.78±1.21 g/dL, respectively (p=0.102). Similarly, no significant difference was detected between cycles with and without pre-infusion hypoalbuminemia regarding pre-infusion platelets with a median of 269.5 x10*^9^/L (range: 126–825) vs. 291 × 10*^9^/L (range: 84–859), respectively, p=0.263.

Regarding hospitalization due to HDMTX-related toxicity, it was more reported in cycles with pre-infusion hypoalbuminemia (39.5%) than those with normal pre-infusion albumin (*p*=0.031). On the other hand, neutropenia, increased ALT, AST, and creatinine were not significantly related to pre-infusion hypoalbuminemia. No significant difference was detected between the two groups when comparing the length of hospitalization due to HDMTX toxicity.

Comparisons of the commonly observed toxicities between 2.5 g/m^2^ HDMTX cycles and 5 g/m^2^ HDMTX cycles are shown in Table 3. Toxicities of HDMTX did not differ significantly between the 2.5 g/m^2^ HDMTX cycles and the 5 g/m^2^ HDMTX cycles except for oral mucositis and length of hospitalization. Oral mucositis was significantly more frequent in the 2.5 g/m^2^ than the 5 g/m^2^ HDMTX cycles (46.7% vs. 26.7%, *p*=0.023). A significantly longer hospitalization (due to HDMTX-related toxicity) was observed in the 5 g/m^2^ HDMTX cycles (median 7 days vs. 4 days, *p*=0.012).

**Table 3:** Comparison of commonly observed toxicities between 2.5 g/m^2^ HDMTX cycles and 5 g/m^2^ HDMTX cycles (*N* = 120)

	2.5 g/m^2^ HDMTX cycles (*N* = 60)	5 g/m^2^ HDMTX cycles (*N* = 60)	*p*
	*N* (%)		
Hematological toxicities			
Neutropenia			
No	16 (26.7)	12 (20.0)	“*χ*^2^ test” *p*=0.388
Yes	44 (73.3)	48 (80.0)	
G3–4	35 (58.3)	35 (58.3)	“*χ*^2^ test” *p*=1.000
Anemia			
No	13 (21.7)	15 (25.0)	“*χ*^2^ test” *p*=0.666
Yes	47 (78.3)	45 (75.0)	
G3–4	17 (28.3)	18 (30.0)	“*χ*^2^ test” *p*=0.841
Thrombocytopenia			
No	39 (65.0)	44 (73.3)	“*χ*^2^ test” *p*=0.323
Yes	21 (35.0)	16 (26.7)	
G3–4	10 (16.7)	9 (15.0)	“*χ*^2^ test” *p*=0.803
Febrile neutropenia^a^			
No	45 (75.0)	44 (73.3)	“*χ*^2^ test” *p*=0.835
Yes	15 (25.0)	16 (26.7)	
Hepatic toxicity			
Increased ALT			
No	46 (76.7)	40 (66.7)	“*χ*^2^ test” *p*=0.224
Yes	14 (23.3)	20 (33.3)	
G3–4	2 (3.3%)	4 (6.7%)	^FE^*p*=0.679
Increased AST			
No	44 (73.3)	43 (71.7)	“*χ*^2^ test” *p*=0.838
Yes	16 (26.7)	17 (28.3)	
G3–4	2 (3.3%)	2 (3.3%)	^FE^*p*=1.000
Gastrointestinal toxicity			
Oral mucositis			
No	32 (53.3)	44 (73.3)	“*χ*^2^ test” *p*=0.023
Yes	28 (46.7)	16 (26.7)	
G3–4	10 (16.7%)	10 (16.7%)	“*χ*^2^ test” *p*=1.000
Nephrotoxicity			
Increased creatinine			
No	41 (68.3)	39 (65.0)	“*χ*^2^ test” *p*=0.699
Yes	19 (31.7)	21 (35.0)	
G3–4	1 (1.7%)	1 (1.7%)	^FE^*p*=1.000
Hospitalization^b^			
No	40 (66.7)	48 (80.0)	“*χ*^2^ test” *p*=0.099
Yes	20 (33.3)	12 (20.0)	
Length of hospitalization	(*N* = 20)	(*N*=12)	
Median (IQR)	4 (2–7.5)	7 (6–10.5)	“*U* test” *p*=0.012
Min.–Max.	2–14	4–12	

*P*-value significant when *p* <0.05

^a^There is no grade 1 or 2 in the CTCAE score system grading febrile neutropenia, and it was reported in the present study only in grade 3 [15]

^b^Extra days of hospitalization due to HDMTX-related toxicities

According to BMI *Z*-scores, 8/30 (26.7%) of children with ALL were malnourished (underweight, overweight, and obese). Pre-infusion hypoalbuminemia was detected in 9/32 (28.1%) HDMTX cycles in the malnourished patients vs. 29/88 (33%) HDMTX cycles in patients with normal BMI. The difference between the two groups was not statistically significant (*p*=0.615).

Moreover, there was no significant difference between malnourished patients and those without malnutrition regarding the commonly observed toxicities in 120 HDMTX cycles (Table 4).

**Table 4:** Comparison between patients with and without malnutrition according to commonly observed toxicities of the HDMTX cycles (*N* = 120)

	Cycles in patients without malnutrition (*N*= 88)	Cycles in patients with malnutrition (*N* = 32)	*p*
	*N* (%)		
Hematological toxicities			
Neutropenia			
No	20 (22.7)	8 (25.0)	“*χ*^2^ test” *p*=0.795
Yes	68 (77.3)	24 (75.0)	
G3–4	49 (55.7)	21 (65.6)	“*χ*^2^ test” *p*=0.329
Anemia			
No	18 (20.5)	10 (31.3)	“*χ*^2^ test” *p*=0.216
Yes	70 (79.5)	22 (68.8)	
G3–4	28 (31.8)	7 (21.9)	“*χ*^2^ test” *p*=0.289
Thrombocytopenia			
No	61 (69.3)	22 (68.8)	“*χ*^2^ test” *p*=0.952
Yes	27 (30.7)	10 (31.3)	
G3–4	15 (17.0)	4 (12.5)	“*χ*^2^ test” *p*=0.546
Febrile neutropenia^a^			
No	66 (75.0)	23 (71.9)	“*χ*^2^ test” *p*=0.729
Yes	22 (25.0)	9 (28.1)	
Hepatic toxicity			
Increased ALT			
No	66 (75.0)	20 (62.5)	“*χ*^2^ test” *p*=0.179
Yes	22 (25.0)	12 (37.5)	
G3–4	5 (5.7%)	1 (3.1%)	^FE^*p*=1.000
Increased AST			
No	68 (77.3)	19 (59.4)	“χ^2^test”*p*=0.052
Yes	20 (22.7)	13 (40.6)	
G3–4	3 (3.4%)	1 (3.1%)	^FE^*p*=1.000
Gastrointestinal toxicity			
Oral mucositis			
No	59 (67.0)	17 (53.1)	“*χ*^2^ test” *p*=0.162
Yes	29 (33.0)	15 (46.9)	
G3–4	14 (15.9%)	6 (18.8%)	“*χ*^2^ test” *p*=0.783
Nephrotoxicity			
Increased creatinine			
No	60 (68.2)	20 (62.5)	“*χ*^2^ test” *p*=0.559
Yes	28 (31.8)	12 (37.5)	
G3–4	1 (1.1%)	1 (3.1%)	^FE^*p*=0.464

*P*-value significant when *p* <0.05

^a^There is no grades 1 or 2 in the CTCAE score system grading febrile neutropenia, and it was reported in the present study only in grade 3 [15]

As for the concomitant drugs given during HDMTX infusion and clearance (for 72 h after the start of HDMTX infusion), antibiotics in the form of penicillin and vancomycin were given in 3/120 (2.5%) and 2/120 (1.7%) of the cycles. Three out of five HDMTX cycles with concomitant penicillin or vancomycin were associated with pre-infusion hypoalbuminemia. The most common HDMTX-related toxicities in these cycles were G3–4 oral mucositis, G3–4 neutropenia, febrile neutropenia, G1–2 anemia, and G1–2 increased creatinine. Each of the ciprofloxacin and proton pump inhibitors was given in 1/120 (0.8%) cycles. None of the enrolled patients experienced extravascular fluid collections that might contribute to MTX delayed elimination such as ascites or pleural effusions.

Plasma MTX levels at 24 h (MTX_24_) were available for the first cycle of consolidation only (30/120 cycles). MTX_24_ ranged from 10.79 to 123.73 μmol/L (median 29.0 μmol/L) in the thirty HDMTX cycles. There was no significant difference in MTX_24_ levels according to MTX dose (2.5 g/m^2^ vs. 5 g/m^2^), with a median of 32.45 μmol/L (range 19.43–73.67) vs. 25.44 μmol/L (range 10.79–123.73), respectively (*p*=0.202). Five out of 15 (33.3%) and 2/15 (13.3%) patients who received 2.5 and 5 g/m^2^ HDMTX had MTX_24_ levels higher than 33 and 65 μmol/L, respectively. Pre-infusion hypoalbuminemia was detected in three patients.

## Discussion

Whereas the survival for pediatric ALL was 10–20% 50 years ago, today᾿s long-term overall survival rates are approximately 80–90% [1]. Much of this improvement in survival can be attributed to developing the most effective chemotherapy therapies, better disease risk stratification, and improved supportive care [2]. The present study’s most commonly encountered toxicities in HDMTX cycles were hematological (neutropenia and anemia), oral mucositis, increased serum creatinine, thrombocytopenia, and increased ALT. These results showed much resemblance to other studies, but with varying frequencies [17, 18]. In the present study, except for oral mucositis and length of hospitalization, toxicities of HDMTX did not differ significantly between the 2.5 g/m^2^ and the 5 g/m^2^ HDMTX cycles. This agrees with other studies [19, 20], confirming that irrespective of the MTX dose, the toxicity of HDMTX was still comparable. The present work was also consistent with other studies reporting significantly reduced rates of oral mucositis with 5 g/m^2^ HDMTX compared to lower HDMTX doses [21, 22]. This finding might be explained by the greater cumulative LV dosages, and the more vigorous pre-hydration procedures used in the 5 g/m^2^ HDMTX when compared to the lower HDMTX doses [21]. On the other hand, hospitalization due to MTX toxicity was significantly longer in the 5 g/m^2^ HDMTX cycles than the 2.5 g/m^2^ HDMTX cycles. This may reflect that the toxicities related to higher HDMTX doses needed a longer time to resolve.

In agreement with the present study results, Reiss et al. [5] had reported that hypoalbuminemia before HDMTX infusion was associated with anemia in adults with leukemia or lymphoma. They also observed that patients with lower albumin levels (≤3.4g/dL) had significantly longer hospitalization.

In our study, anemia, thrombocytopenia, febrile neutropenia, and oral mucositis were significantly more frequent in HDMTX cycles with pre-infusion hypoalbuminemia when compared to cycles with normal pre-infusion albumin. Furthermore, pre-infusion hypoalbuminemia was found to be associated with G3–4 anemia and G3–4 thrombocytopenia. There was no significant difference in pre-infusion hemoglobin levels or platelets between cycles with and without hypoalbuminemia. The higher toxicity rates with hypoalbuminemia could be explained by the fact that low albumin levels are associated with delayed clearance of MTX with a potential increase in the risk of toxicity [23].

On the other hand, no significant relationship could be detected between pre-infusion hypoalbuminemia with G3–4 neutropenia, hepatotoxicity, or nephrotoxicity. Wiczer et al. [24] investigated variables that may contribute to methotrexate-induced renal toxicity in adults receiving HDMTX for treatment of leukemia or lymphoma, and they observed that low serum albumin (<3 g/dL) was a significant risk factor for nephrotoxicity. This difference may be due to the higher cutoff value of albumin used in our study.

Recognized drug interactions may delay MTX clearance and increase toxicity. Drugs that delay MTX renal secretion through possible blockage of proximal transporters are mainly involved in interactions that have a major clinical impact (toxicity) and should be avoided. Penicillin, NSAIDs, salicylic acid, and proton pump inhibitors are examples of these medications [25]. The presence of concomitant drugs during HDMTX infusion and clearance in the present study was only reported in 7/120 (5.8%) of all HDMTX cycles. So, we were not able to assess drug–drug interactions and the possible potentiation of HDMTX toxicity.

The MTX_24_ level is particularly relevant for monitoring after HDMTX as it reflects the steady-state concentration during infusion. Kataoka et al. [23] who investigated 74 adults receiving HDMTX recommended MTX_24_ monitoring in patients with low albumin levels (<3.7 g/dL) to detect high-peak MTX concentrations that occurred as a result of delayed clearance. According to the total XV protocol, target 24-h MTX steady-state plasma concentrations are 33 and 65 μmol/L for the low risk and the standard/high-risk arms, respectively [13]. In the present study, 5/15 (33.3%) and 2/15 (13.3%) of patients who received HDMTX at 2.5 g/m^2^ and 5 g/m^2^ had higher MTX_24_ levels than the targeted for this MTX dose. Three out of the seven high MTX_24_ levels were associated with pre-infusion hypoalbuminemia. Reiss et al. [5] compared time to MTX clearance (defined as the first documented time the MTX level ≤0.05 μmol) between patients with normal albumin levels and patients with hypoalbuminemia and found that hypoalbuminemia was significantly associated with longer MTX clearance time (median 96 vs. 72 h, *p*=0.004).

Malnutrition (undernutrition, overweight, and obesity) in children with cancer is associated with overall worse outcomes from diagnosis to long-term survival [8]. In the current study, 2/30 (6.7%), 4/30 (13.3%), and 2/30 (6.7%) of children were underweight, overweight, and obese, respectively. These percentages are higher than that reported in a multi-center study conducted by the Middle East Childhood Cancer Alliance [26], where 0.5% and 3.1% of children with ALL were underweight and obese at diagnosis, respectively. Nevertheless, the BMI *Z*-scores calculated in the present study were based on weights measured after steroid therapy in the induction phase.

Seki et al. [27] observed that weight gain is often observed in children with ALL who undergo chemotherapeutic treatment, including steroids. Although it may be thought a transient phenomenon, Withycombe et al. [28] found that the increase of BMI *Z*-scores during induction was an independent predictor of obesity at the end of therapy. A recent study identified obesity and large size as new risk factors for delayed MTX elimination in patients aged 10.2 to 19.2 years old treated with HDMTX [29]. Interestingly, a study showed a negative association between obesity and albumin level among children [30]. In the present study, there was no significant relationship between hypoalbuminemia and the nutritional status of patients indicated by BMI *Z*-scores. In addition, there were no significant differences in HDMTX-related toxicities between malnourished patients and those without malnutrition. These contradicting results may be due to the small number of malnourished ALL children enrolled in the present study.

The present study highlights the importance of optimizing serum albumin levels of children once recognized as future HDMTX candidates. A nutrition specialist should be involved early in the multi-disciplinary team treating children with ALL. Raising serum albumin can be gradually achieved by applying a proper nutritional plan to avoid potential HDMTX toxicities [24]. Other strategies of rapidly correcting serum albumin levels using human serum albumin are not encouraged. The majority of infused human serum albumin is redistributed into the extravascular space within 48 h, and therefore, it would be costly, temporary, and of uncertain benefit [5].

On the other hand, the present study had some limitations. First, we had a relatively small sample size, having only 38 cycles with hypoalbuminemia. Second, plasma MTX levels could not be estimated in all cycles and at more time points post-HDMTX infusion due to the unavailability of the test at our center and its high cost.

## Conclusion

In conclusion, pre-infusion hypoalbuminemia (serum albumin <3.5 g/dL) was significantly associated with more frequent HDMTX-related toxicities. Our findings suggest that before starting HDMTX treatment in any cycle, serum albumin levels should be checked, especially in resource-limited settings where malnutrition is highly prevalent, and serum MTX monitoring may not be available in a timely manner. Optimizing serum albumin for HDMTX candidates may help decrease the frequency of possible HDMTX toxicities.

## Data Availability

The datasets used and/or analyzed during the current study are available from the corresponding author on reasonable request.

## Abbreviations

ALL: Acute lymphoblastic leukemia
MTX: Methotrexate
IV: Intravenous
HDMTX: High-dose methotrexate
hr: Hour
LV: Leucovorin
MTX_24_ level: Methotrexate level at 24 h
ELISA: Enzyme-linked immunosorbent assay
BMI: Body mass index
WHO: World Health Organization
SD: Standard deviation
CTCAE: Common Terminology Criteria for Adverse Events
ALT: Alanine transferase
AST: Aspartate transaminase
G: Grade
Vs: Versus
*N*: Number
%: Percent
IQR: Interquartile range
Min.-Max.: Minimum–maximum
p: *P* value
*χ*^2^: Chi-square test
^FE^p: *P* value for Fishers’ exact test
U: Mann-Whitney test

## References

[1] Lee JW, Cho B. Prognostic factors and treatment of pediatric acute lymphoblastic leukemia. Korean J Pediatr. 2017;60(5):129–37. 28592975 10.3345/kjp.2017.60.5.129 PMC5461276

[2] Hunger SP, Mullighan CG. Acute lymphoblastic leukemia in children. N Engl J Med. 2015;373(16):1541–52. 26465987 10.1056/NEJMra1400972

[3] Levêque D, Becker G, Toussaint E, Fornecker LM, Paillard C. Clinical pharmacokinetics of methotrexate in oncology. Int J Pharm. 2017;2(2):137–47.

[4] Kotur N, Lazic J, Ristivojevic B, Stankovic B, Gasic V, Dokmanovic L, et al. Pharmacogenomic markers of methotrexate response in the consolidation phase of pediatric acute lymphoblastic leukemia treatment. Genes. 2020;11:1–17. 10.3390/genes11040468 PMC7230684 32344632

[5] Reiss SN, Buie LW, Adel N, Goldman DA, Devlin SM, Douer D. Hypoalbuminemia is significantly associated with increased clearance time of high dose methotrexate in patients being treated for lymphoma or leukemia. Ann Hematol. 2016;95(12):2009–15. 27542957 10.1007/s00277-016-2795-7 PMC5572815

[6] McLean TW, Stewart RM, Curley TP, Dewsnup MY, Thomas SG, Russell TB, et al. Hypoalbuminemia in children with cancer treated with chemotherapy. Pediatr Blood Cancer. 2020;67(2):e28065. 31736232 10.1002/pbc.28065 PMC6939630

[7] Schmiegelow K, Rank CU, Stock W, Dworkin E, van der Sluis I. SOHO state of the art updates and next questions: management of asparaginase toxicity in adolescents and young adults with acute lymphoblastic leukemia. Clin Lymphoma Myeloma Leuk. 2021;21(11):725–33. 34511319 10.1016/j.clml.2021.07.009

[8] Diakatou V, Vassilakou T. Nutritional status of pediatric cancer patients at diagnosis and correlations with treatment, clinical outcome and the long-term growth and health of survivors. Children (Basel). 2020;7(11):218. 33171756 10.3390/children7110218 PMC7694979

[9] Ge KY, Chang SY. Definition and measurement of child malnutrition. Biomed Environ Sci. 2001;14(4):283–91. 11862608

[10] Martín-Trejo JA, Núñez-Enríquez JC, Fajardo-Gutiérrez A, Medina-Sansón A, Flores-Lujano J, Jiménez-Hernández E, et al. Early mortality in children with acute lymphoblastic leukemia in a developing country: the role of malnutrition at diagnosis. A multicenter cohort MIGICCL study. Leuk Lymphoma. 2017;58(4):898–908. 27561220 10.1080/10428194.2016.1219904

[11] Tandon S, Moulik NR, Kumar A, Mahdi AA, Kumar A. Effect of pre-treatment nutritional status, folate and vitamin B12 levels on induction chemotherapy in children with acute lymphoblastic leukemia. Indian Pediatr. 2015;52(5):385–9. 26061923 10.1007/s13312-015-0642-x

[12] Kanbayashi Y, Nomura K, Okamoto K, Matsumoto Y, Horiike S, Takagi T, et al. Statistical examination to determine whether only 48-h value for serum concentration during high-dose methotrexate therapy is a predictor for clinical adverse events using ordered logistic regression analysis. Ann Hematol. 2010;89(10):965–9. 20425113 10.1007/s00277-010-0965-6

[13] Pui CH, Relling MV, Sandlund JT, Downing JR, Campana D, Evans WE. Rationale and design of Total Therapy Study XV for newly diagnosed childhood acute lymphoblastic leukemia. Ann Hematol. 2004;83(Suppl 1):S124–6. 15124703 10.1007/s00277-004-0850-2

[14] WHO Multicentre Growth Reference Study Group. WHO Child Growth Standards based on length/height, weight and age. Acta Paediatr. 2006;450:76–85. 10.1111/j.1651-2227.2006.tb02378.x 16817681

[15] Common Terminology Criteria for Adverse Events (CTCAE), Version 4.03, June 14, 2010. US Department of Health and Human Services. National Institutes of Health National Cancer Institute.

[16] Lo SF. Reference intervals for laboratory tests and procedures. In: Kliegman RM, Geme III JW.ST, Blum NJ, Shah SS, Tasker RC, Wilson KM, et al. editors. Nelson Textbook of Pediatrics. 21th Philadelphia:Saunders Elsevier; 2019. p. 14795-14808.

[17] Li X, Sui Z, Jing F, Xu W, Sun S, Guo Q, et al. Identifying risk factors for high-dose methotrexate-induced toxicities in children with acute lymphoblastic leukemia. Cancer Manag Res. 2019;11:6265–74. 31308758 10.2147/CMAR.S207959 PMC6615715

[18] Mandal P, Samaddar S, Chandra J, Parakh N, Goel M. Adverse effects with intravenous methotrexate in children with acute lymphoblastic leukemia/lymphoma: a retrospective study. Indian J Hematol Blood Transfus. 2020;36(3):498–504. 32647424 10.1007/s12288-019-01245-z PMC7326748

[19] Vaishnavi K, Bansal D, Trehan A, Jain R, Attri SV. Improving the safety of high-dose methotrexate for children with hematologic cancers in settings without access to MTX levels using extended hydration and additional leucovorin. Pediatr Blood Cancer. 2018;65(12):e27241. 29768710 10.1002/pbc.27241

[20] Khera S, Kapoor R, Pramanik SK. Solitary serum methotrexate level 36 hours post high-dose methotrexate: a safe, efficacious and cost-effective strategy to monitor methotrexate toxicities in childhood leukemia in resource-limited centers. Pediatr Blood Cancer. 2020:e28387. 10.1002/pbc.28387 32400952

[21] Van der Beeka JN, Oosteroma N, Pietersa R, de Jongec R, van den Heuvel-Eibrinka MM, Heil SG. The effect of leucovorin rescue therapy on methotrexate-induced oral mucositis in the treatment of paediatric ALL: a systematic review. Crit Rev Oncol Hematol. 2019;142:1–8. 31323533 10.1016/j.critrevonc.2019.07.003

[22] Xu W, Tang Y, Song H, Shi S, Yang S. Retrospective study on elimination delay of methotrexate in high-dose therapy of childhood acute lymphoblastic leukemia in China. J Pediatr Hematol Oncol. 2007;29(10):688–93. 17921849 10.1097/MPH.0b013e31814d6777

[23] Kataoka T, Sakurashita H, Kajikawa K, Saeki Y, Taogoshi T, Matsuo H. Low serum albumin level is a risk factor for delayed methotrexate elimination in high-dose methotrexate treatment. Ann Pharmacother. 2021;55(10):1195–202. 33543634 10.1177/1060028021992767

[24] Wiczer T, Dotson E, Tuten A, Phillips G, Maddocks K. Evaluation of incidence and risk factors for high-dose methotrexate-induced nephrotoxicity. J Oncol Pharm Pract. 2016;22(3):430–6. 26152702 10.1177/1078155215594417

[25] Levêque D, Santucci R, Gourieux G, Herbrecht R. Pharmacokinetic drug-drug interactions with methotrexate in oncology. Exp Rev Clin Pharmacol. 2011;4:743–50. 10.1586/ecp.11.57 22111860

[26] Al-Mulla NA, Chandra P, Khattab M, Madanat F, Vossough P, Torfa E, et al. Childhood acute lymphoblastic leukemia in the Middle East and neighboring countries: a prospective multi-institutional international collaborative study (CALLME1) by the Middle East Childhood Cancer Alliance (MECCA). Pediatr Blood Cancer. 2014;61(8):1403–10. 24648275 10.1002/pbc.25031

[27] Seki Y, Okamoto Y, Kodama Y, Nishikawa T, Tanabe T, Nakagawa S, et al. Risk factors and the prevention of weight gain during induction chemotherapy in children with acute lymphoblastic leukemia. J Pediatr Hematol Oncol. 2018;40(6):e334–7. 29401101 10.1097/MPH.0000000000001098

[28] Withycombe JS, Smith LM, Meza JL, Merkle C, Faulkner MS, Ritter L, et al. Weight change during childhood acute lymphoblastic leukemia induction therapy predicts obesity: a report from the Children's Oncology Group. Pediatr Blood Cancer. 2015;62:434–9. 25407299 10.1002/pbc.25316 PMC4304977

[29] Orgel E, Nabais T, Douglas C, Mittelman SD, Neely M. Effect of body fat on population pharmacokinetics of high-dose methotrexate in pediatric patients with acute lymphoblastic leukemia. J Clin Pharmacol. 2021;61(6):755–62. 33314168 10.1002/jcph.1799

[30] Nishimura R, Kanda A, Sano H, Matsudaira T, Miyashita Y, Morimoto A, et al. Glycated albumin is low in obese, non-diabetic children. Diabetes Res Clin Pract. 2006;71(3):334–8. 16154660 10.1016/j.diabres.2005.07.008

